# Malignant ascites-derived exosomes promote proliferation and induce carcinoma-associated fibroblasts transition in peritoneal mesothelial cells

**DOI:** 10.18632/oncotarget.15040

**Published:** 2017-02-02

**Authors:** Mingtian Wei, Tinghan Yang, Xiangzheng Chen, Yangping Wu, Xiangbing Deng, Wanbin He, Jinliang Yang, Ziqiang Wang

**Affiliations:** ^1^ Department of Gastrointestinal Surgery, West China Hospital, Sichuan University, Chengdu, Sichuan 610041, China; ^2^ State Key Laboratory of Biotherapy and Cancer Center/Collaborative Innovation Center for Biotherapy, West China Hospital, West China Medical School, Sichuan University, Chengdu, Sichuan 610041, China; ^3^ Department of Liver Surgery and Liver Transplantation Center, West China Hospital, Sichuan University, Chengdu, Sichuan 610041, China

**Keywords:** exosomes, mesothelial cells, peritoneal metastasis, proliferation, carcinoma-associated fibroblasts

## Abstract

Malignant ascites-derived exosomes have been demonstrated to participate in tumor metastasis. In peritoneal metastasis, normal mesothelial cells (MCs) can be converted into carcinoma-associated fibroblasts (CAFs) by mesothelial-mesenchymal transition (MMT). Herein, we evaluated the effect of malignant ascites-derived exosomes on peritoneal MCs *in vitro* and *in vivo* experiments to determine whether exosomes could educate MCs and contribute to peritoneal metastasis.

Under the treatment of ascites-derived exosomes, peritoneal MCs showed increased ability to proliferate and migrate. Expression of CAFs specific proteins markers in MCs, including fibroblast activation protein (FAP), alpha-smooth muscle actin (α-SMA), and fibronectin, were increased after treatment of exosomes. In clinical samples test, TGF-β1 was found to be overexpressed in both malignant ascites and malignant ascites-derived exosomes, and the high volume of TGF-β1 may be responsible for peritoneum fibrosis. In addition, exosomes can increase xenograft tumor growth by suppressing the inhibitive ability on tumor cells by MCs. Besides, CAFs specific proteins markers including FAP, α-SMA, and vimentin were increased in clinical peritoneal biopsies. The immunohistochemical staining for mice tumor biopsies also revealed increased expression of fibronectin and FAP, along with decreased expression of E-cadherin and VCAM-1 after exosomes treatment.

Thus, malignant ascites-derived exosomes may be of importance in the development of peritoneal metastasis by facilitating MCs to proliferate and convert into CAFs by TGF-β1 induced MMT.

## INTRODUCTION

Exosomes are late endosomes-derived membrane vesicles with size ranging from 30 to 120 nm in diameter [1, 2]. To date, exosomes are reported to be secreted by most malignant and normal cells, distributing in numerous bodily fluids, such as plasma, urine, saliva, breast milk, and malignant effusions [3, 4]. As a star sub-cellular structure, tumor-derived exosomes display crucial roles in intercellular communications not only between tumor and stromal cells, but also tumor and distant target cells or microenvironments, by selectively delivering its cargoes (membrane or inner proteins, DNAs, and mRNAs) [5]. Recently, emerging evidences have validated that exosomes are somewhat responsible for tumor metastasis. For instance, pancreatic cancer-derived macrophage migration inhibitory factor (MIF)^+^ exosomes facilitate liver metastasis [6], and exosomal integrin α6β4 and α6β1 are associated with lung metastasis [7].

In tumor metastatic stroma, carcinoma-associated fibroblasts (CAFs) are the prominent composition cell types which are involved in solid tumor seeding, angiogenesis, and formation of pre-metastatic niche to facilitate cancer cell dissemination [8]. The origin of CAFs differs in various tumors and specific sites of individual tumors. Although the origin of CAFs is obscure, resident fibroblasts account for the one origin of CAFs and other cells such as epithelial cells, mesenchymal cells, endothelial cells can also be the source of CAFs [9]. In mechanism, transforming growth factor beta 1 (TGF-β1) mainly induces the conversion to CAFs via epithelial-mesenchymal transition (EMT), which facilitates the migration and invasion of tumor cells.

In normal peritoneum, a single layer of mesothelial cells (MCs) and subjacent resident fibroblasts, monocytes, mast-cells, capillary, and lymph vessels compose the structure of peritoneal membrane. When inflammatory factors and other agents are released into the ascites in tumor environment or damage response, the first contact MCs may antagonize the attack by proliferating, transition, or altering the phenotypes. In peritoneal metastasis, a recent research has certified that normal MCs can be converted into CAFs by ascites-derived soluble TGF-β1 induced mesothelial-mesenchymal transition (MMT), which is characterized by an increased migration and invasion capacity of MCs [10]. Since peritoneal metastasis is usually accompanied by malignant ascites, which are enriched with exosomes, and exosomes have TGF-β1 as its cargo, it is intriguing to speculate that exosomes from malignant ascites could educate peritoneal MCs. However, the impact of exosomes on MCs has not been investigated.

In the current study, we focused on malignant ascites-derived exosomes, revealing their impact on MCs *in vitro* experiments and *in vivo* model. These results, along with clinical samples validation, showed strong connections between exosomes and peritoneal metastasis. Therefore, we propose that malignant ascites-derived exosomes induce MCs transition into CAFs to facilitate peritoneal metastasis.

## RESULTS

### Exosomes identification

**Figure 1:**
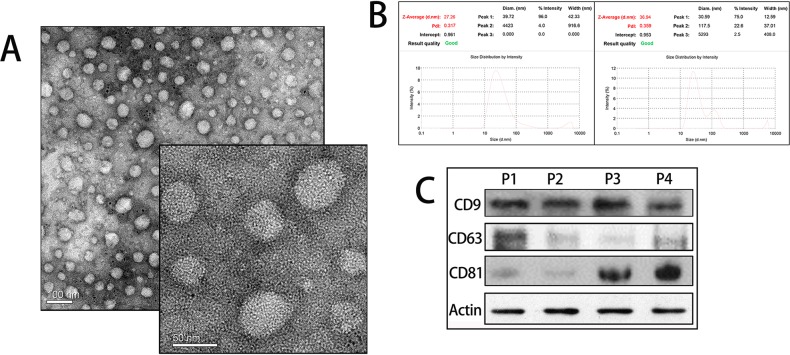
Identification of exosomes **A**. TEM images of exosomes. Exosomes were defined as round shaped membrane vesicles in size 30-100nm. **B**. Size distribution of exosomes decided by DLS. Left exosomes from malignant ascites and right exosomes from culture media. **C**. Western blot analysis of exosomal markers. P1, P2 represented gastric cancer exosomes and P3, P4 represented ovarian cancer exosomes.

To identity the exosomes, we applied three different techniques: Transmission Electron Microscopy (TEM), Dynamic light scattering (DLS), and Western Blot analysis for exosomes’ specific markers. Malignant ascites-derived exosomes were lightly stained with diameters within 30-100 nm under TEM (Figure 1A). For DLS, the size distribution was assessed using Malvern Instruments. The result of DLS method was shown in Figure 1B. Cell-derived exosomes showed two distinct peaks at 30.59 nm and 117.5 nm with Z-Average (d. nm) 36.94 and malignant ascites-derived exosomes showed a single peak at 39.72 nm with Z- Average (d.nm) 27.26 (Figure 1B). Western Blot revealed four malignant ascites-derived exosomes (two gastric cancers and two ovarian cancers). CD9, CD63, and CD81, which are exosomes-specific markers, were detectable in all samples (Figure 1C).

### The uptake of exosomes by MCs

**Figure 2:**
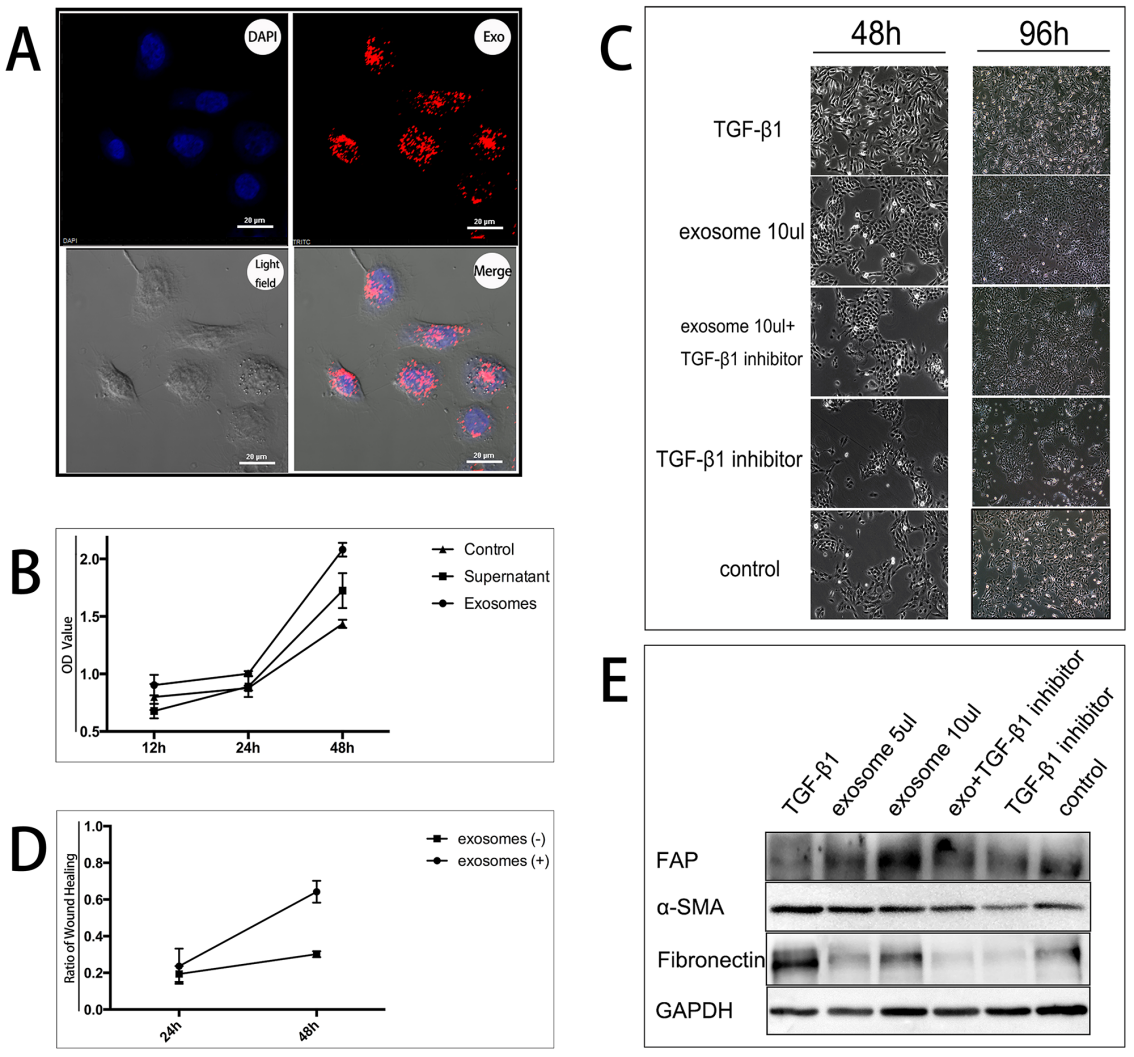
Exosomes promote HMrSV5 cells transition into CAFs by proliferation *in vitro* **A**. The uptake of tumor-derived exosomes by MCs. HMrSV5 cells incubated with PKH26 labeled tumor-derived exosomes for overnight. The upper left panel shows DAPI-dyed HMrSV5 cell nucleus. The upper right panel shows PKH26-dyed exosomes. The lower left panel shows light field image of HMrSV5 cells. The lower right panel shows merge image demonstrating PKH26 labeled exosomes entered HMrSV5 cells. **B**. Proliferation of HMrSV5 cells after treatment with exosomes. HMrSV5 cells at 5×10^3^ cells/well were treated with exosomes for 12, 24 and 48 h. HMrSV5 cells treated with the same amount of DMEM served as control. CCK-8 assays were performed in triplicate for each value. After 48 h, the number of HMrSV5 cells treated by exosomes (represented by OD value) was significantly higher than those without treatment p < 0.05. **C**. 5/10 μl exosomes (10 μg/105 PBL) or 1 μl TGF-β1 (5 ng/ml) promoted proliferation of HMrSV5 cells under morphological observation. Adding 0.76 μl TGF-β1 inhibitor (10 μM/L) alleviated the effect of exosomes or TGF-β1. **D**. Wound healing ratio of HMrSV5 cells after scratch closure assay. After 48 h, wound healing ratio (recovered region/initial scratched region) of HMrSV5 cells treated by exosomes was significantly higher than rate of non-treated cells p < 0.05. **E**. Western blotting assay of FAP, α-SMA and fibronectin in HMrSV5 cells treated by exosomes or TGF-β1.

After overnight incubation with labeled exosomes, numerous HMrSV5 acquired positive PKH26 signal viewed by a confocal microscope (Figure 2A). Adding dye alone produced no intracellular incorporation (Supplementary Figure 1). This finding revealed the incorporation of tumor-derived exosomes with MCs *in vitro*.

### Exosomes enhanced proliferation of MCs

The effect of exosomes on HMrSV5 cells was investigated. HMrSV5 cells were seeded on 96 well plate dishes and treated with exosomes for different periods of time. Exosomes effectively induced proliferation in HMrSV5 cells in a time-dependent manner (Figure 2B). Morphologically, HMrSV5 cells grew into more spindle-like after exosomes or TGF-β1 positive treatments for 48 and 96 hours, compared with that in TGF-β1 inhibitor or blank control treatments (Figure 2C).

To further confirm the proliferative effect of exosomes, a scratch closure test was performed. After 48 h, the width of the scratch was measured, and was found to be smaller in the exosomes-treated HMrSV5 cells than in the non-treated (Supplementary Figure 2). The scratch closure test demonstrated that the recovered region was increased with exosomes treatment (Figure 2D). The recovered region/initial scratched region or wound healing ratio was larger in the samples treated with exosomes than in the non-treated samples, indicating that the ability to preliferate and migrate was enhanced by treating with exosomes.

### Exosomes promote HMrSV5 cells transition into CAFs by proliferation *in vitro*

To explore the underlying mechanism of proliferation of HMrSV5 cells after treatment of malignant ascites-derived exosomes, we assumed HMrSV5 cells may undergo phenotype conversion. Western blot analyzed three CAFs-specific proteins markers fibroblast activation protein (FAP), alpha-smooth muscle actin (α-SMA), and fibronectin. All expression level of proteins were increased after exosomes treatment compared with control. At the same time, a corresponding increase was also observed in TGF-β1 treatment and the expression level was decreased by TGF-β1inhibitor (Figure 2E). Previous study showed TGF-β1 as a key promoter for CAFs transition [11]. Exosomes treatment seemed to have a similar result with sole TGF-β1 treatment, showing the possible pattern of exosomes function driven by TGF-β1. Exosomes-treated MCs proliferated faster and presented spindle-like morphology, consistent with shape of CAFs. Briefly, malignant ascites-derived exosomes may induce HMrSV5 cells conversion into CAFs by its cargo TGF-β1.

### Malignant ascites-derived exosomes overexpress TGF-β1 and may induce peritoneum fibrosis

**Figure 3:**
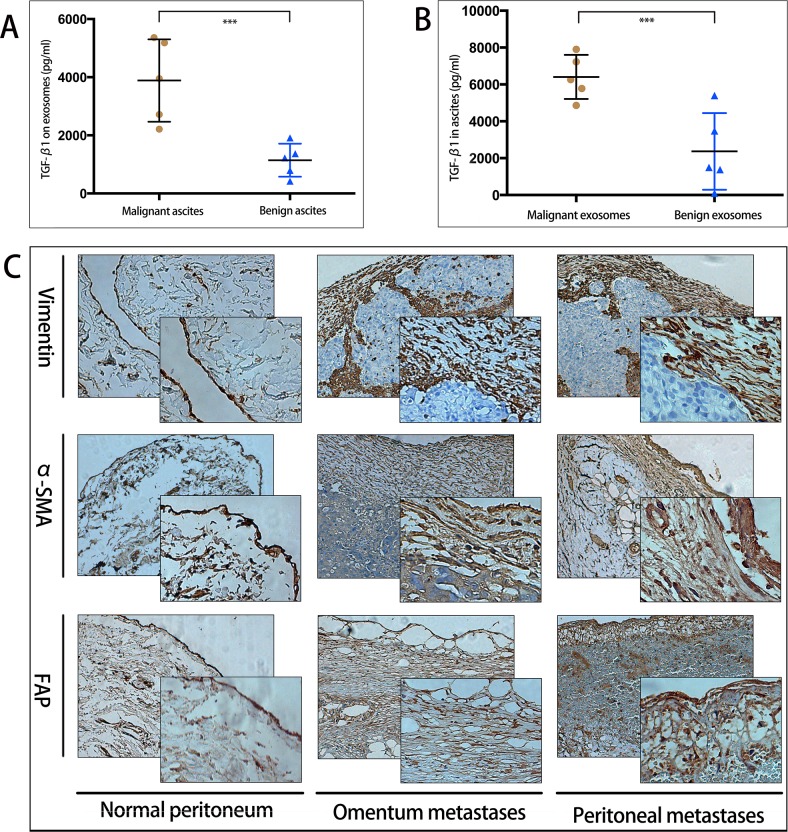
Malignant ascites-derived exosomes overexpress TGF-β1 and may induce peritoneum fibrosis **A**. Expression level of TGF-β1 (pg/ml) in free malignant and benign ascites, ascites from 10 patients (5 malignant tumors with peritoneal metastasis, 5 hepatic cirrhosis), *** indicates statistical difference. **B**. Expression level of TGF-β1 (pg/ng) on malignant and benign ascites-derived exosomes, the same patients as in **A**, *** indicates statistical difference. **C**. IHC analysis of fibrosis markers of peritoneal biopsies. Left panel indicated the normal peritoneum with single layer MCs. Middle and right panels both revealed overexpression of CAFs markers vimentin, α-SMA, FAP in omentum metastasis and peritoneal metastasis.

Clinically, malignant and benign ascites were obtained to extract exosomes. TGF-β1 was detected highly expressed both in malignant ascites and in malignant ascites-derived exosomes, compared with those in benign ascites (Figure 3A, 3B). To illustrate the potiential impact of exosomal TGF-β1 on peritonum, we further measured the fibrosis of peritonum and omentum by immunohistochemical (IHC) staining. Compared to normal peritonum biospies which represented single layer MCs, peritoneal and omental metastases biospies showed significantly incresed fibrosis with expressed CAFs markers CAFs-specific proteins markers FAP, α-SMA, and vimentin (Figure 3C). These results together indicated increased level of exosomes in ascites may correlate to CAFs formation.

### MCs treated by ascites-derived exosomes regulate xenograft tumor growth *in vivo*

**Figure 4:**
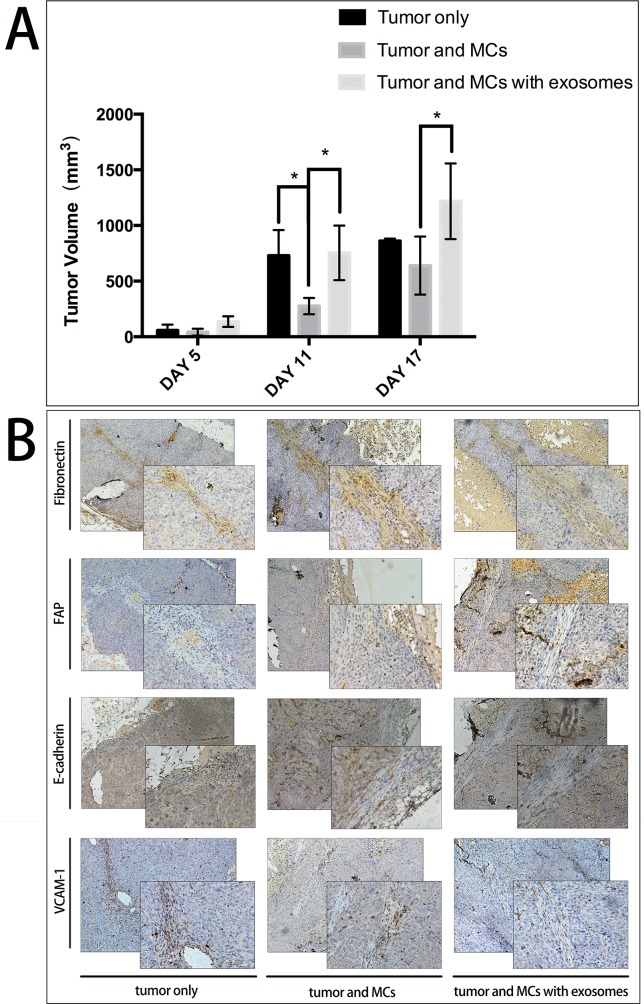
MCs treated by tumor-derived exosomes promote xenograft tumor growth **A**. Bar chart of xenograft tumor sizes in three groups: tumor representatives treated with HCT116 cells only, HCT116 cells and MCs, HCT116 cells with MCs and exosomes in nude mice. **B**. IHC analysis of EMT markers of mice tumor biopsies. Left panel indicated the expression of fibronectin, FAP, E-cadherin, and VCAM-1 in group with HCT116 cells only. Compared to this group, middle and right panels revealed increased expression of fibronectin and FAP proteins, and decreased expression of E-cadherin and VCAM-1 proteins in the other two groups.

To evaluate whether MCs treated by tumor-derived exosomes can regulate tumor growth, MKN45 cells (1 × 10^7^) and MCs (2 × 10^5^) treated by exosomes were injected subcutaneously in BALB/c nude mice. After 11 days, we observed that tumor size in those mice injected with MCs were significantly smaller than in other groups. In the mean time, tumor size in those mice injected with exosomes-treated MCs were comparable to tumor size in those mice injected with tumor cells only (Figure 4A). On day 14, tumors in 3 out of 5 mice injected with tumor cells only and 4 out of 5 mice injected with exosomes-treated MCs developed hemorrhagic necrosis while no mouse injected with non-treated MCs developed the same symptoms. To minimize further suffering from hemorrhagic necrosis, all mice were sacrificed on day 17. IHC staining of mice tumor biopsies revealed increased expression of fibronectin and FAP, and decreased expression of E-cadherin and VCAM-1 after exosomes treatment (Figure 4B). Other three markers including cytokeratin, collagen I, and α-SMA did not change (Supplementary Figure 3).

## DISCUSSION

Two existing theories, up to now, have been proposed to elucidate peritoneal metastases. Except for “milky spots” pathway in omentum majus, which may be the fisrt pathway for detached tumor cells seeding to form metastases. Peritoneal mesothelium is the alternative pathway for free tumor cells to colonize [12, 13]. Physiologically, the monolayer of MCs maintains the intact structure of peritoneum and protects itself from inflammatory invasion. However, if the microenvironment around peritoneum is well induced, as the “seed and soil” theory goes, the peritoneum becomes a suitable “soil” for tumor cells “seeding” and growth. The MCs loss their protective function and alters their phenotypes. In peritoneal metastases process, although emerging evidences have verified the MMT transition of mesothelium by TGF-β1 factor, no publications report the similar effect of tumor-derived exosomes [14–17]. Herein, our study for the first time demonstrated the transition of mesothelium induced by exosomes.

Briefly, the present study firstly demonstrated that tumor-derived exosomes could be internalized by MCs and enhance proliferation of these cells *in vitro*. Next, we observed the phenotype change of MCs under morphology and Western blot test, which both indicated CAFs-like transition. Finally, vivo model and clinical spacemen indirectly demonstrate the positive connection of high volume of exosomes and peritoneal metastases, which was also mediated by CAFs transition. Accumulatively, this study proposed a novel function of tumor-derived exosomes on MCs by promoting proliferation and inducing MCs into CAFs.

Previous researches have demonstrated TGF-β1 as a crucial factor to induce EMT transition to form CAFs. Fuyuhiro et al. showed gastric fibroblasts underwent CAFs conversion by TGF-β induction in scirrhous gastric cancer [18]. And other studies also have also found the similar results of TGF-β1 on inducing CAFs formation [11, 19–21]. In our design, we set TGF-β1 as the positive control, which acquires the similar effect on mesothelium with exosomes treatment. Thus, we proposed that exosomes may function throughout its membrane anchor protein such as TGF-β1. In fact, exosomes have been reported to mediate intercellular communication by delivering its proteins and micro-RNAs. A recent study confirmed a novel mechanism that hepatocyte-derived exosomes carry neutral ceramidase and sphingosine kinase 2 to target cells to facilitate proliferation [22]. Similarly, glypican-1 specifically enriched on pancreatic cancer-cell-derived exosomes was deemed as a novel diagnostic and prognostic protein [23]. These findings, along with ours, emphasize the important role of tumor-derived exosomes and their likely functions substances in cellular communication. In addition, a recent study have shown that CAFs were found in peritoneal metastatic implants derive from the mesothelium through a mechanism TGF-β1-induced MMT of MCs, forming a suitable metastatic niche to promote cancer adhesion/invasion and growth [10]. Thus, We assumed that tumor-derived exosomes at least partly mediated the process of MMT.

Besides, in our experiment, we have roughly revisited how the exosomes transfer messages to target cells. Using PKH26 labeling, we located the exact position of exosomes in the cytoplasm after co-incubation with HMrSV5 in Figure 2A. That is to say, tumor-derived exosomes may be firstly internalized by target cells as the fundamental step to work. Like classical pathways of delivering functional cargo, exosomes could be recognized and absorbed by recipient cells in both endocytosis and phagocytosis manners. Hazan-Halevy and his colleagues observe the uptake of mantle cell lymphoma-derived exosomes specifically by B lymphocytes in a lipid raft endocytosis pathway [24]. And another research demonstrates that exosomes internalization into cell cytoplasm is dependent of ERK1/2-HSP27 signaling, also by a lipid raft-mediated endocytosis manner [25]. In this process, a few adhesion proteins were elevated to promote the internalization of exosomes, however, without clear mechanism. Our findings, although lacking detailed molecular mechanism, are in line with that in previous studies that exosomes are internalized to function. This mechanism of uptake may provide a useful insight for blocking up potential ill communication between cell–cell or cell–matrix mediated by exosomes.

Instestingly, in our *in vivo* model, we found the MCs act as a defender of tumor cells’ implatation, indicating its anti-metastasis function in normal physiology. Exosomes antagonised the protection of MCs and facilitate the metastasis of free tumor cells, especially *in vivo* fluid ascites. This result, on the other hand, verified the hypothesis that exosomes may induce the MCs transform into CAFs to promote metastasis. Although MCs have been reported to prevent cancer invasion in normal physiology, our findings on the interaction between tumor-derived exosomes and MCs in regulating peritoneal metastasis is novel, which we are establishing the detailed connection *in vitro* experiments.

One weakness in our present study is the failure to validate where and how exosomes work with MCs *in vivo* model. Although exosomes have been certified to facilitate metastasis of tumor cells, however, we lack the direct evidences of impact of tumor-derived exosomes on MCs, as we have observed in the vitro experiments. Another limitation is mechanism of exosomes involved in the transition by TGF-β1 induced MMT, if there are other proteins or RNAs participating in this transition is unknown.

In conclusion, our work shows that tumor-derived exosomes may be of importance in the development of peritoneal metastasis by facilitating MCs to proliferate and convert into CAFs by TGF-β1 induced MMT. Prospective studies to reveal the connection between exosomes and MCs *in vivo* are recommended.

## MATERIALS AND METHODS

### Cell culture

The human peritoneal mesothelial cell line HMrSV5 was kindly provided by Prof. Youming Peng of the Second Hospital, Zhongnan University, Changsha, China. This cell line was established after infection of a fully characterized primary culture of human peritoneal MCs with an amphotropic recombinant retrovirus that encodes the SV40 Large-T Ag under the control of the Moloney virus long terminal repeat as previously described [26]. Human gastric cancer cell line MKN45 and ovarian cancer cell line SKOV3 were purchased from the cell bank of the Chinese Academy of Science (Shanghai, China). HMrSV5 cells were cultured in RPMI1640 and cancer cells were cultured in DMEM at 37°C in a humidified atmosphere of 5% CO_2_. All culture media contained 10% FBS, 100 U/mL of penicillin and 100μg/mL of streptomycin. For subsequent exosomes isolation, cancer cells were cultured for 48 h and then the culture media were changed to serum-free DMEM after washing with PBS for three times. Cancer cells were cultured for another 24 h before further use.

### Exosomes isolation

Exosomes from the culture medium and malignant ascites were isolated by Exo-Quick Exosome Precipitation Solution (System Biosciences, CA, USA) according to the manufacturer's instructions.

Exosomes pellets were resuspended with the appropriate volume of the serum-free medium or PBS. Exosomes from 1 × 10^6^ cells or 10 mL malignant ascites were suspended in 150 μl of medium or PBS.

### Transmission electron microscopy (TEM)

The pelleted exosomes were resuspended with PBS and diluted at 1:100-1000. Then 20 μl of diluted exosome was dripped on a copper grid gently. After air drying at room temperature for 3 minutes, 3% phosphotungstic acid was added to dye exosomes for 5 minutes. The samples were then observed under a TecnaiG2F20 FE-TEM (Philips, Amsterdam, Netherlands).

### Western blot

Cell line proteins were extracted as previously reported [27]. Total proteins were quantified by Nanodrop™ 2000/2000c spectrophotometers (Thermo-Fisher Scientific, Waltham, Massachusetts, USA). Equal amounts of denatured proteins (usually 20μg) were fractionated by 8% or 10% sodium dodecyl sulphate polyacrylamide gel (SDS-PAGE) electrophoresis, and transferred to a polyvinylidene difluoride (PVDF) membrane. The membrane was blocked with 5% non-fat milk at 37°C for 2 hours, and subsequently incubated with a primary antibody with a diluted concentration based on its protocol overnight at 4°C. After washing three times in Tris-buffered saline with Tween 20 (TBST) in 10 minutes’ interval, the blots were then incubated with a peroxidase-conjugated secondary antibody at 37°C for 1.5 hours. Finally, after washing as before, the blots were detected using an enhanced chemiluminescence system (Amersham Biosciences, Buckinghamshire, UK). β-Actin or GAPDH were used as an internal control.

### Dynamic light scattering (DLS)

DLS is a commonly used technique to estimate the size of small particles. We applied DLS (Malvern Instruments, Worcestershire, UK) to assess the size distribution of exosomes. Exosomes were obtained from the supernatants of culture media or malignant ascites. Then exosomes size was measured using DLS method. The analyzer presented the size distribution of exosomes graphically as intensity plots.

### PKH26 labelling and analysis

Exosomes were labeled with the red fluorescent dye PKH26 (Sigma-Aldrich, St. Louis, Missouri, USA) according to the manufacturer's protocol. The stained exosomes were then ultracentrifuged at 100,000 × g for 2 h, washed and resuspended with PBS. Subsequently, exosomes were added to HMrSV5 cells in culture in 170 μm Cell Imaging Dishes (Eppendorf, Hamburg, Germany). After overnight incubation and three washing steps with 1 × PBS, HMrSV5 cells were air dry, fixed with 4 % paraformaldehyde and stained with DAPI (Dojindo, Kumamoto, Japan). A Laser scanning confocal microscope (Nikon, Japan) was used to observe the uptake of exosomes.

### Proliferation assay

Cell Counting Kit-8 (CCK-8, Dojindo, Kumamoto, Japan) assay was conducted to identify the effect of exosomes on HMrSV5 cells. For this purpose, 5.0 × 10^3^ HMrSV5 cells were seeded on 96 well plate dishes and allowed to proliferate for 48 h. The samples were treated with 50 μg mL^−1^ of exosomes for 12 h, 24h or 48 h. The cultured media were aspirated and the CCK-8 mixed media (1:10 - CCK-8: DMEM) were added to the samples. After 2 h, the absorbance of CCK-8 mixed media at 450 nm was measured using a spectrophotometer (Bio-Rad, Hercules, California, USA). Three cultures were exposed to each solution for each time period.

### Scratch closure test

HMrSV5 cells were cultured until confluent on the 6 well plate dishes and the cell monolayer was scraped by a pipette tip in a straight line. The detached cells and debris were removed by washing with PBS buffer. Subsequently, exosomes were added to the scratched monolayer. Images of the scratched area were taken after 48 h by phase-contrast microscopy (Olympus, Shinjuku, Tokyo, Japan). To evaluate wound closure, the widths of the scratched area were measured with microscopic images.

### Biopsies and ascites from clinical patients

Both peritoneal and omental metastases from one gastric cancer and four ovarican cancers were intra-operatively obtained. Normal peritoneum from two retacl cancers, three colon cancers, and two benign abdominal tumors were acquired as controls. Besides, malignant (two ovarian, one pancreatic, one hepatic, and one rhabdomyosarcoma cancers) and benign (four hepatic cirrhosis and one tuberculosis) ascites samples were collected. All patients signed informed written consent with the approval of the Biological and Medical Ethics Committee of West China Hospital. Tissue samples were fixed in 4% formalin immediately after acquisition, and embedded in paraffin to obtain serial sections 3-5μm thick. Haematoxylin and eosin (HE), along with IHC staining were performed, as described below. Ascites were centrifuged at 2000 g for 30 minutes at room temperature to remove cells and debris and then stored at −80°C for further use.

### Immunohistochemical analysis of clinical tissues and nude mice tissues

Deparaffinized tissues were heated to expose the antigens using Tris-ethylene diamine tetraacetic acid (EDTA) retrieval solution (pH 6.0) at 95°C for 30 minutes. Wash with PBS for three times per 3 minutes and incubate the sections in 3% hydrogen peroxide (H_2_O_2_) to block endogenous peroxidase activity. And then, samples were stained using primary antibodies to detect α-SMA (Abcam, ab32575), FAP (Abcam, ab28244), vimentin (Abcam, ab92547), fibronectin (Abcam, ab45688), E-cadherin (Abcam, ab76319), VCAM-1 (Abcam, ab134047), collagen I (Abcam, ab138492), cytokeratin (Abcam, ab6401). Antibodies were followed by means of peroxidase-conjugated secondary antibody and revealed with 3-39-diaminobenzidine (DAB) as chromogen.

### *In vivo* study

For *in vivo* assay, the MKN45 cells (1 × 10^7^) and/or MCs (2 × 10^5^) treated by exosomes were suspended in 200 μl PBS and then injected subcutaneously into right side of the posterior flank of five BALB/c nude mice. Tumor growth was examined daily, and the tumor volumes were calculated every week using the formula for hemi-ellipsoids: V = length (cm) × width (cm) × width (cm) × 0.52. After 3 weeks, each mouse was sacrificed, and the tumors were dissected and weighed. Animals experiment for this research was designed and carried out according to the standard guideline of Institutional Animal Care and Use Committee (IACUC), and the study design had been approved by Institutional Animal Care and Use Committee.

### Statistical analysis

Statistical analysis was performed using SPSS 22.0 for mac (IBM Corporation, Armonk, NY, USA). For continual variables, all data were evaluated using Student's *t*-test. For all analyses, a p value less than 0.05 was considered significant.

## SUPPLEMENTARY MATERIALS FIGURES AND TABLES


