# Supplementary material for: Malignant ascites-derived exosomes promote proliferation and induce carcinoma-associated fibroblasts transition in peritoneal mesothelial cells
Source: Oncotarget. 2017 Feb 2;8(26):42262–71. doi: 10.18632/oncotarget.15040 (PMC5522065; doi:10.18632/oncotarget.15040)
Supplement: Supplementary file 1 [file oncotarget-08-42262-s001.pdf]

Malignant ascites-derived exosomes promote proliferation and induce carcinoma-associated fibroblasts transition in peritoneal mesothelial cells

SUPPLEMENTARY FIGURES

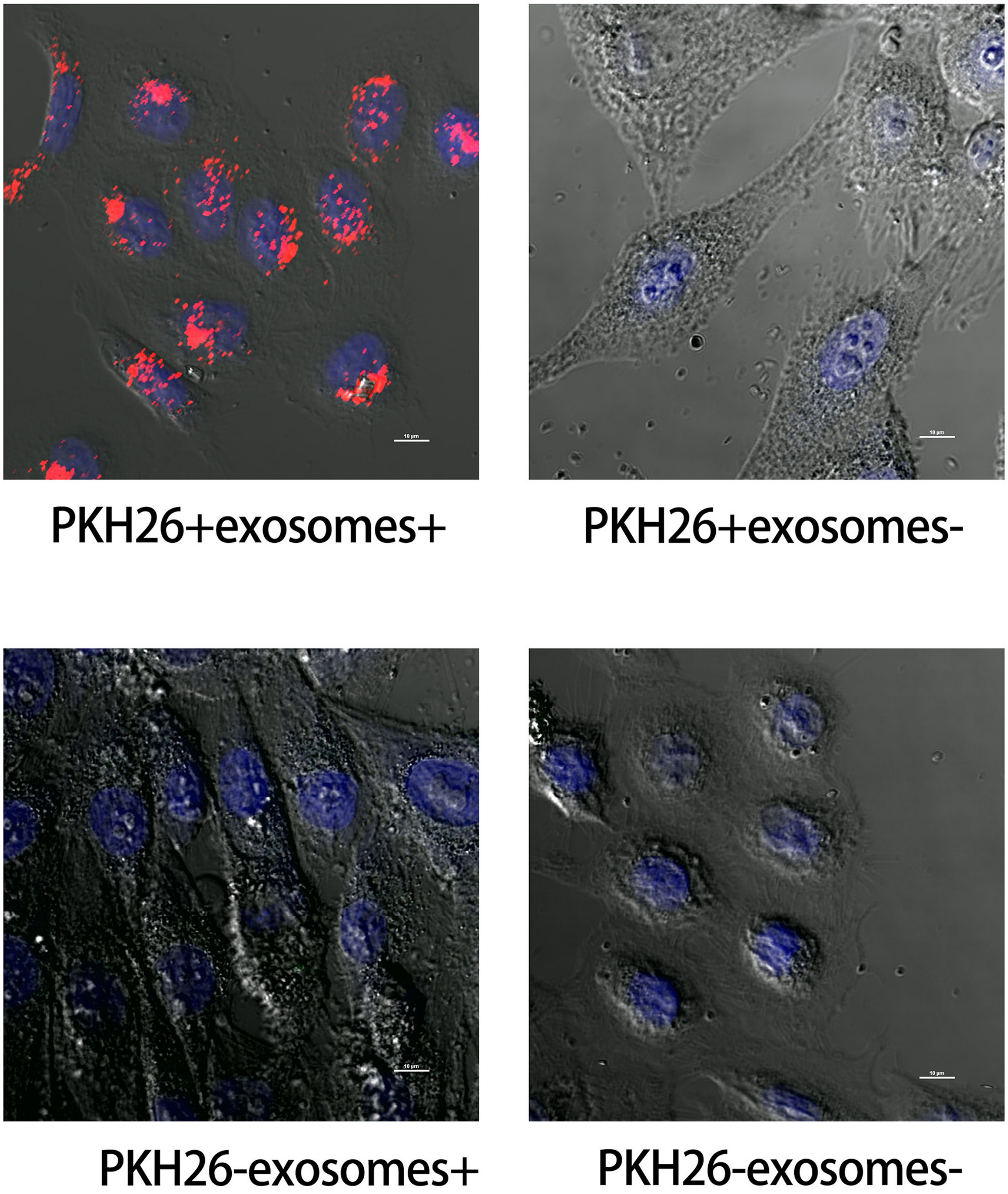

Supplementary Figure 1: Uptake of tumor-derived exosomes by HMrSV5 cells.

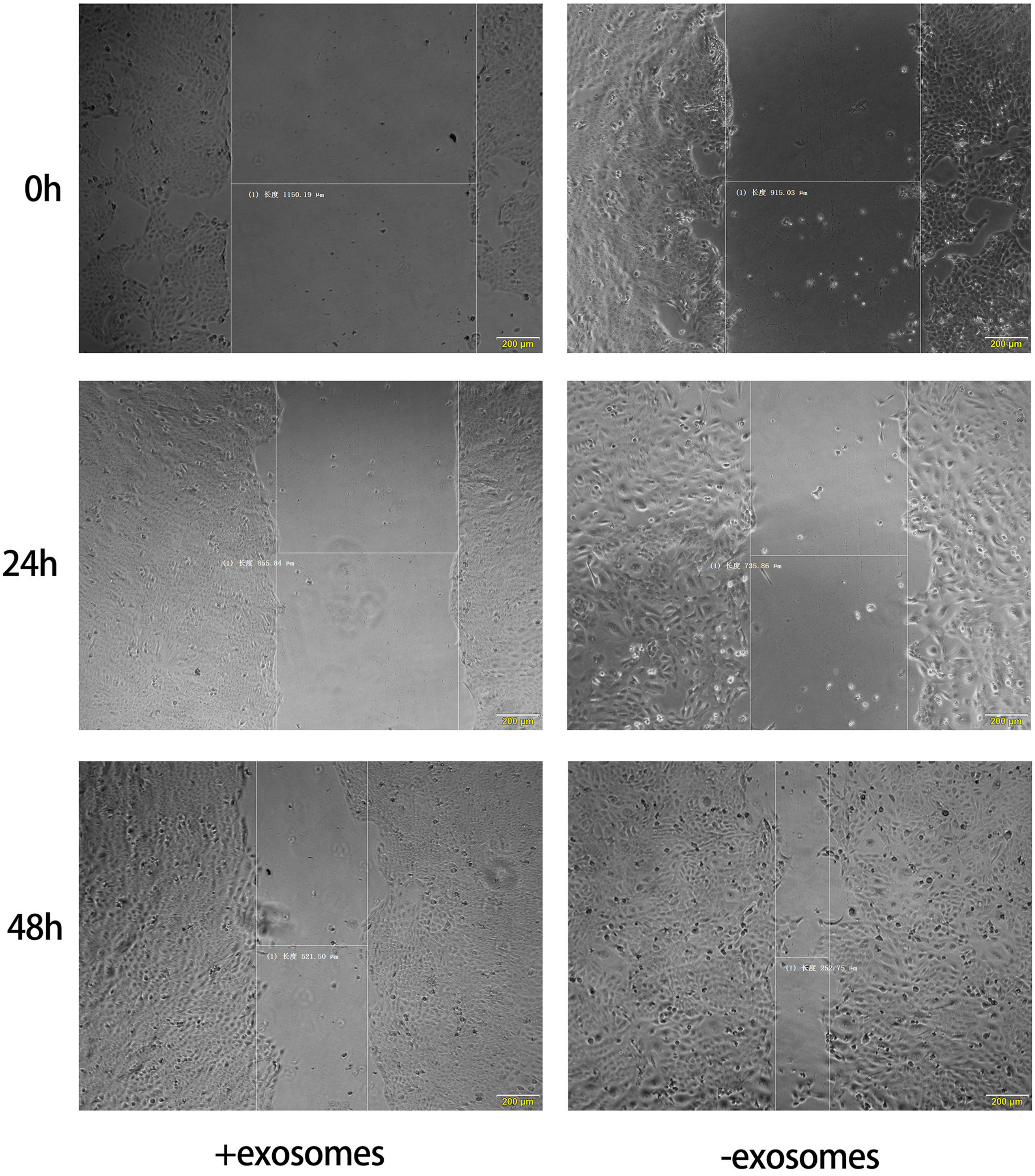

Supplementary Figure 2: Effects of tumor-derived exosomes on HMrSV5 cells using a scratch closure assay.

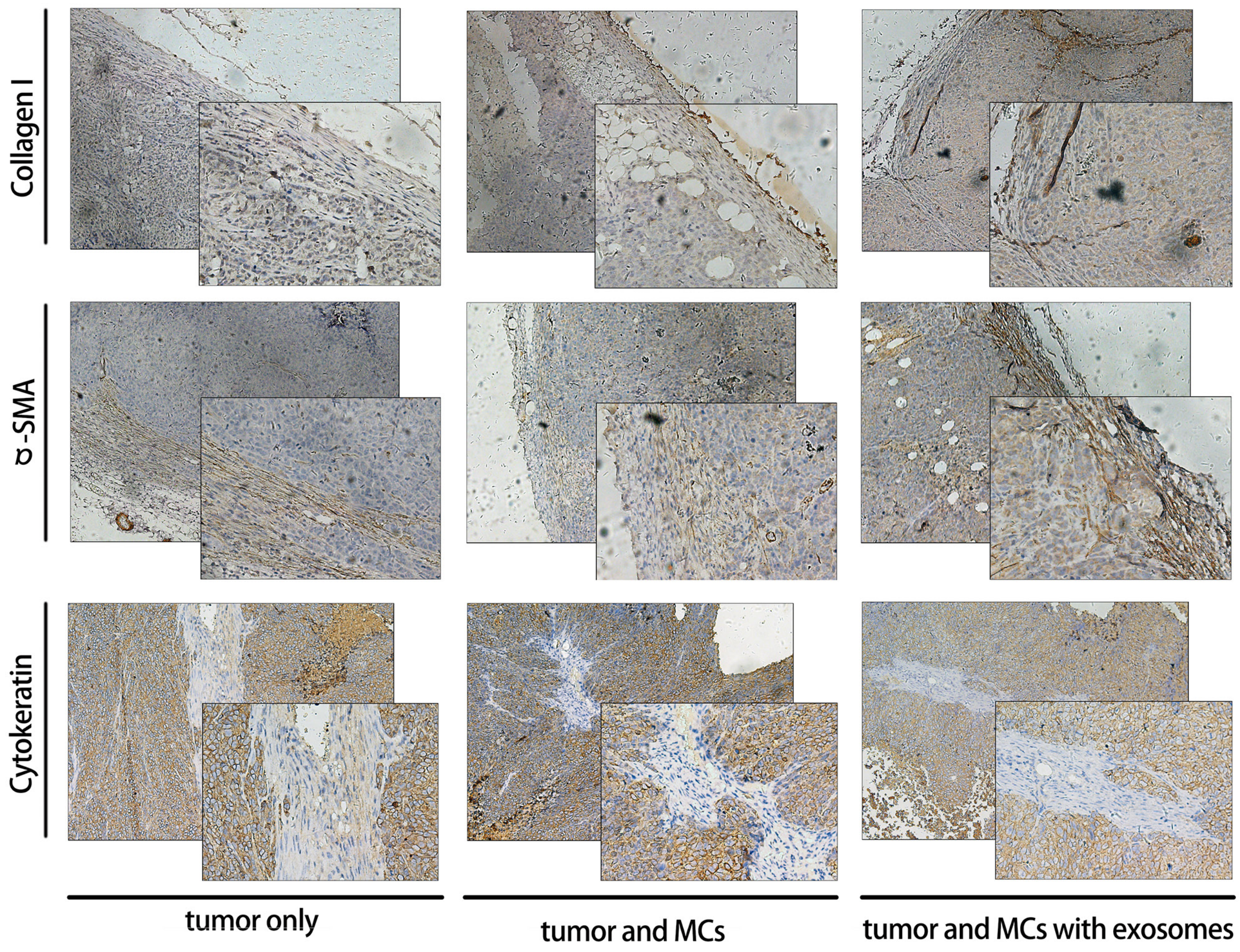

Supplementary Figure 3: IHC analysis of EMT markers (cytokeratin, α-SMA, and collagen I) of mice tumor biopsies.
